# Supplementary material for: Development of the CRISPR-Cas9 System for the Marine-Derived Fungi Spiromastix sp. SCSIO F190 and Aspergillus sp. SCSIO SX7S7
Source: J Fungi (Basel). 2022 Jul 8;8(7):715. doi: 10.3390/jof8070715 (PMC9322911; doi:10.3390/jof8070715)
Supplement: Supplementary file 1 [file jof-08-00715-s001.zip › jof-1784553-supplementary.pdf]

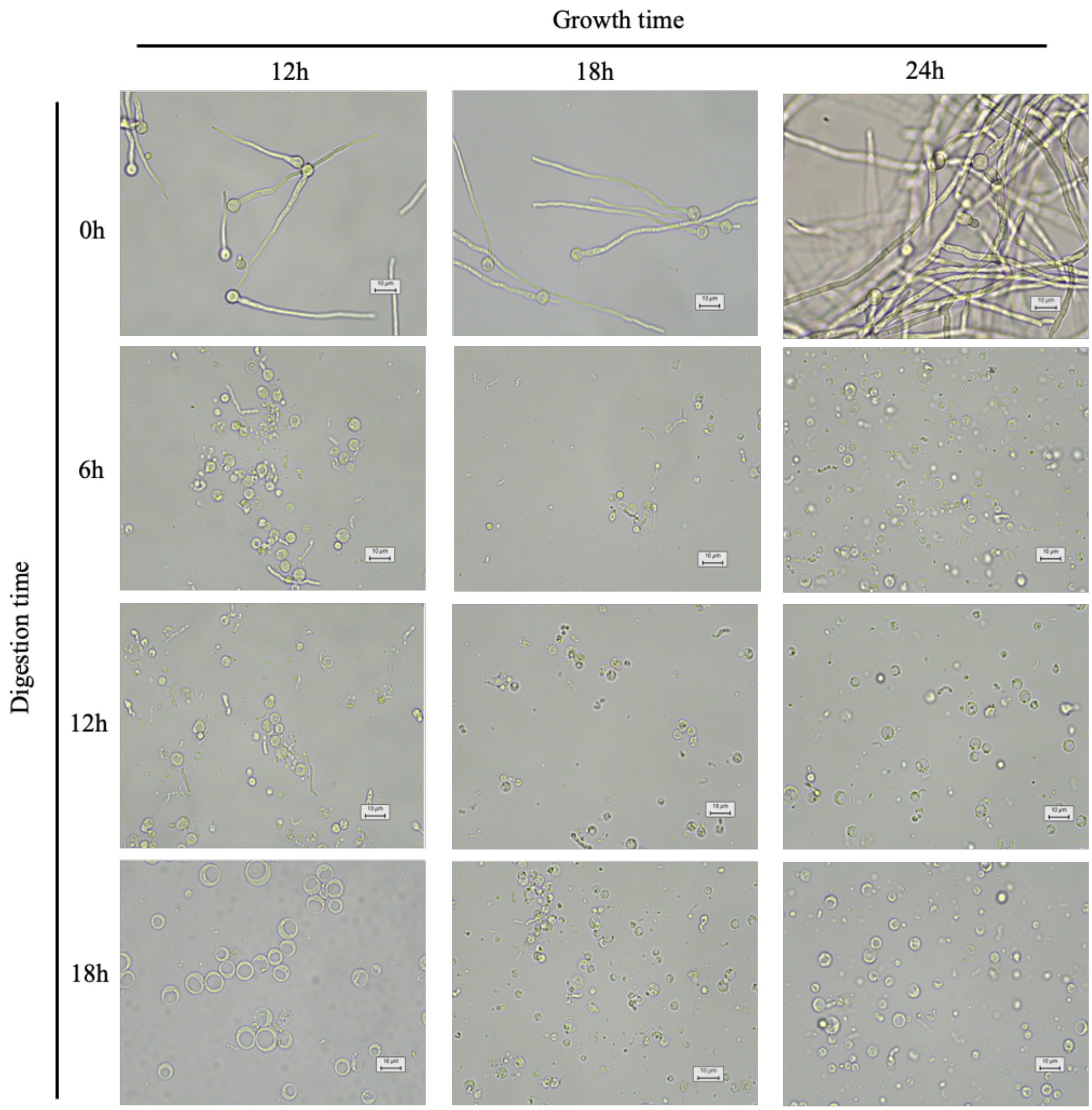

Figure S1. Microscopic check of germination and protoplast released from the mycelium of *Aspergillus* sp. SCSIO SX7S7 under different times of culture and enzyme digestion.

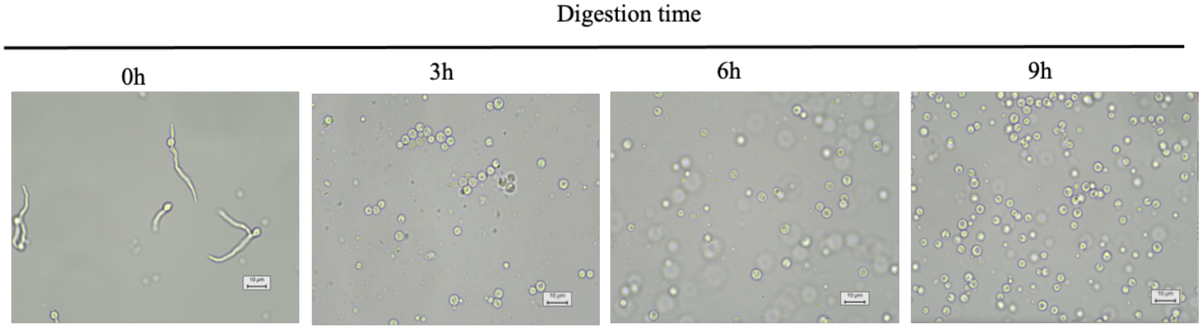

Figure S2. Microscopic check of germination and protoplasts released from the mycelium of *Spiromastix* sp. SCSIO F190 under different times of enzyme digestion.

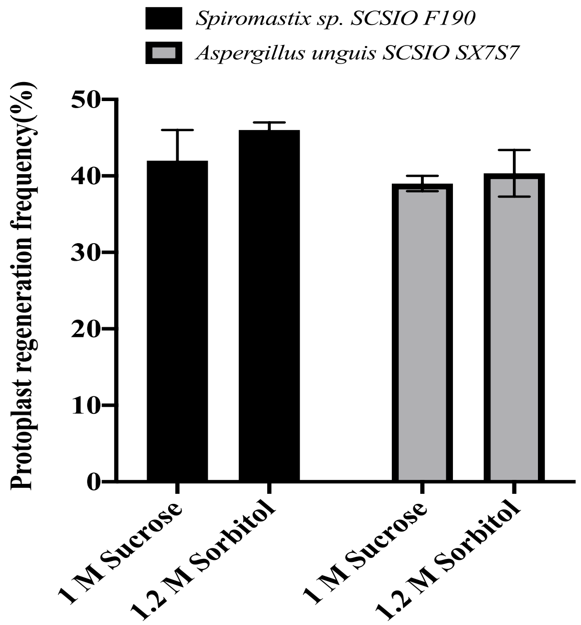

Figure S3. Effects of osmotic pressure stabilizer on *Spiromastix* sp. SCSIO F190 and *Aspergillus* sp. SCSIO SX7S7 protoplast regeneration rate.

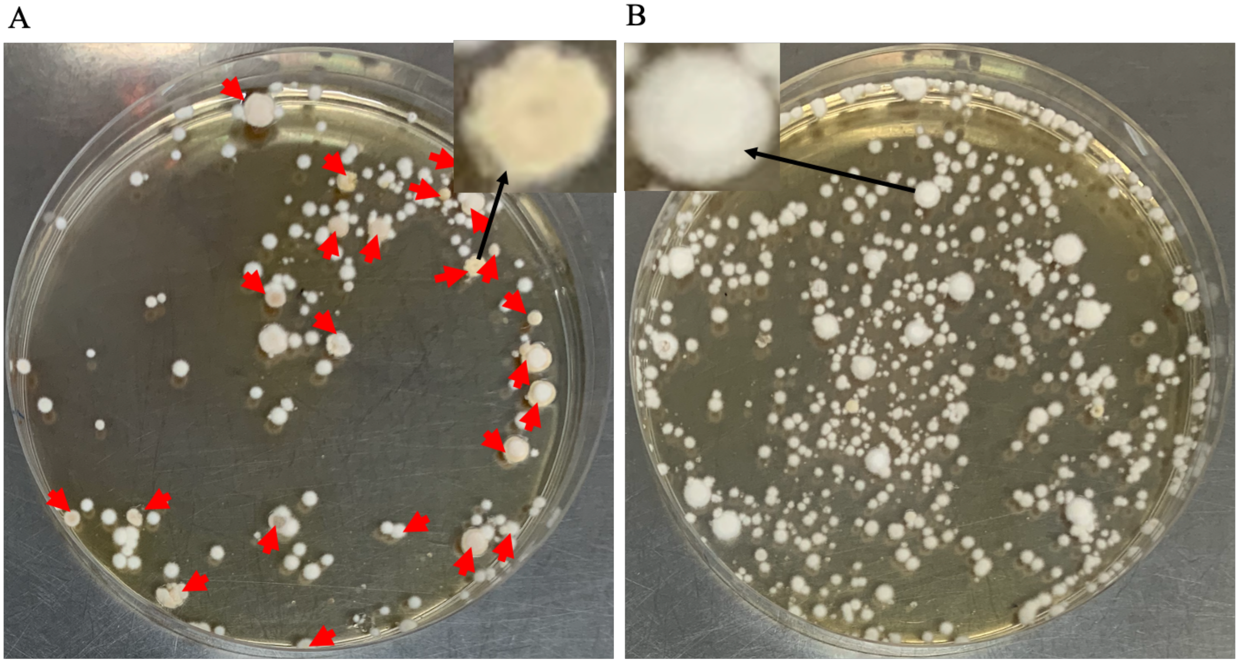

Figure S4. Inactivation of the *creA* gene in *Spiromastix* sp. SCSIO F190 using CRISPR-Cas9. Regeneration plates of transformants with the CRISPR-Cas9 plasmids containing either a sgRNA gene with a protospacer targeting exon of *creA* (A), or no sgRNA gene (B). CRISPR-Cas9 plasmid containing a sgRNA targeting *creA* results in growth defect colonies that accumulated more pigment. The arrows indicate *creA* mutants, and the growth phenotype of representative clones were showed in amplified figure.

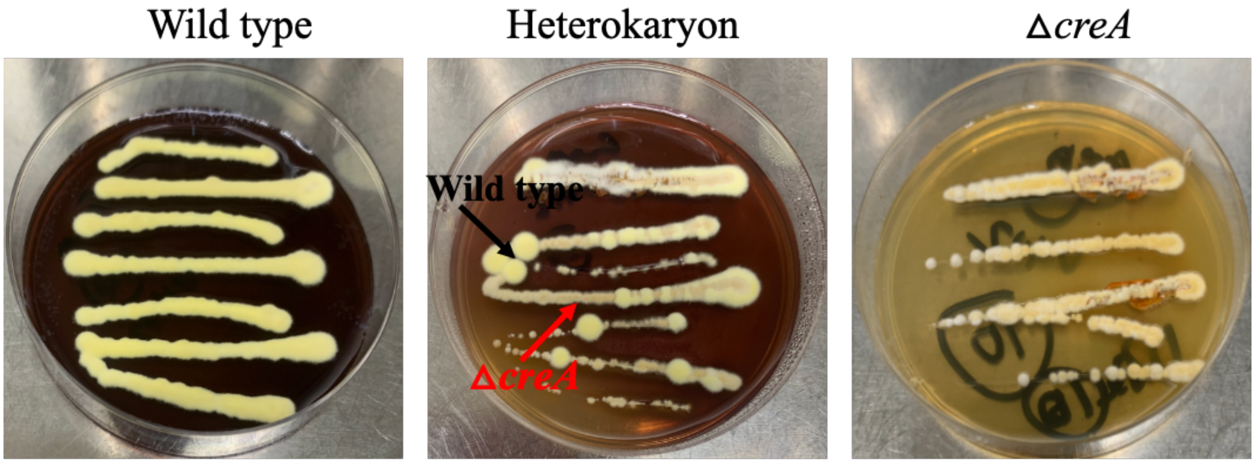

Figure S5. Morphological comparison of the Δ*creA* and wild type heterokaryotic strain with *Spiromastix* sp. SCSIO F190 wild type strain and the pure Δ*creA* mutant. All strains were grown on PDA plate at 28 °C for 14 days.

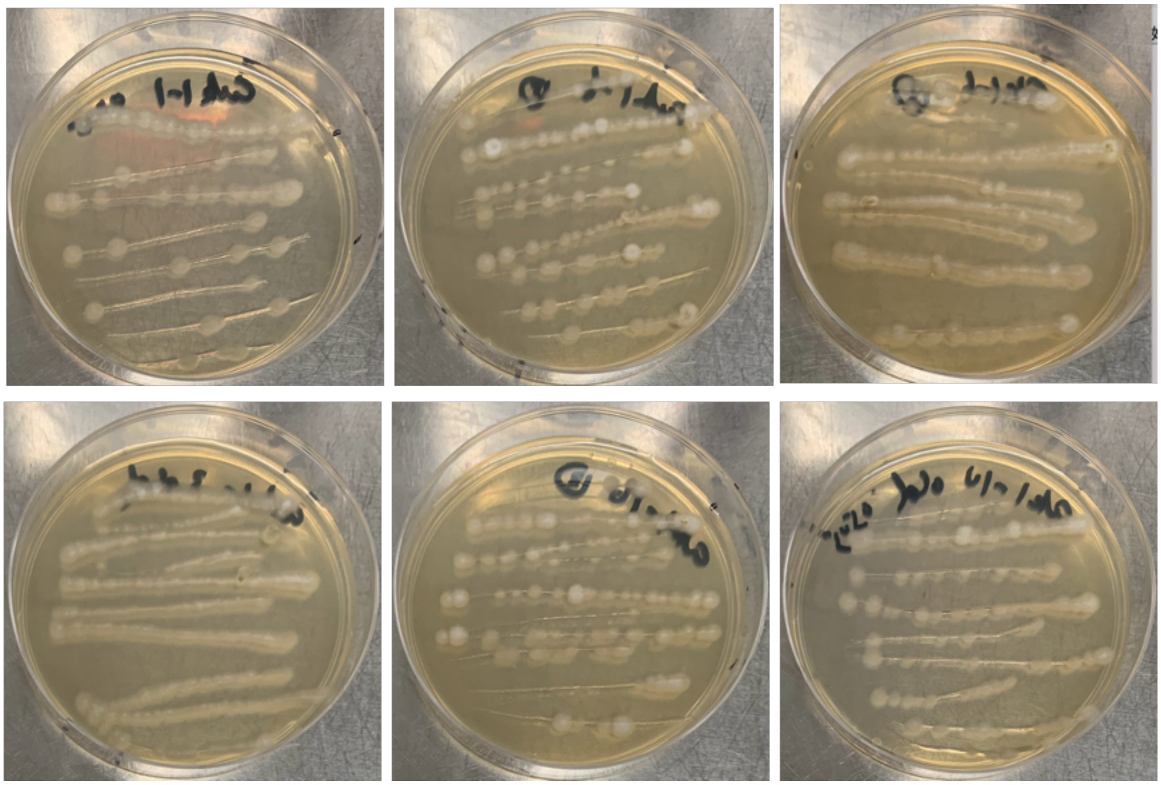

Figure S6. Phenotype of the selected Δ*cak1* mutants in *Aspergillus* sp. SCSIO SX7S7 grown on PDA plate for 4 days at 28 °C.

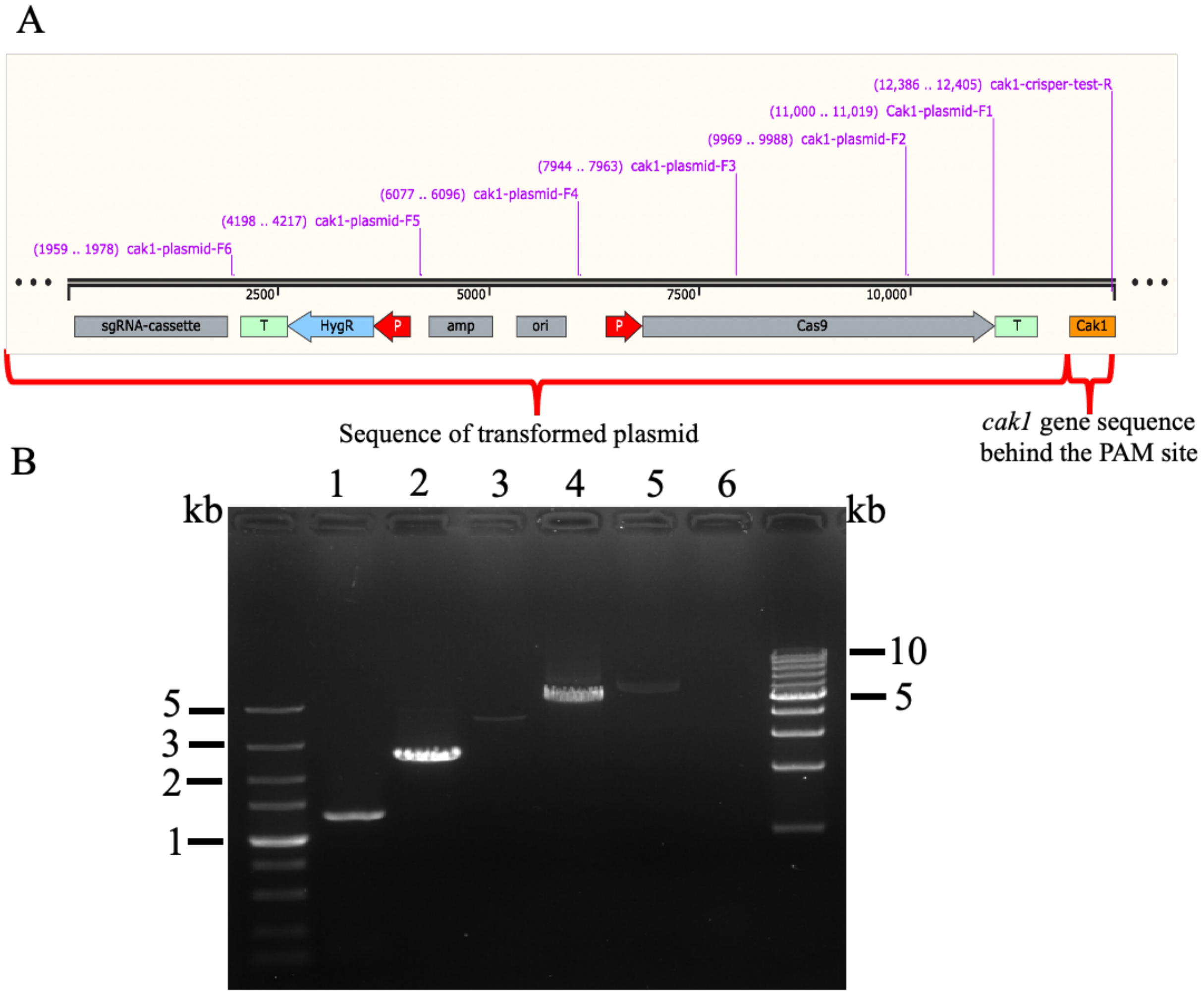

Figure S7 PCR verification of the inserted pBSKII-to*Cas9*-*hph*-sgRNA plasmid sequence into *cak1* gene. To test the validity of the insertion sequence, DNA amplifications were performed across the insert-genomic DNA junction using flanking genomic DNA primer "R" and vector primers "F1-6" (A) . PCR products with expected sizes were observed except for a product with length larger than 10kb (B), indicating a contiguous vector was inserted at the target site. Lane 1-6: DNA amplifications of vector-*cak1* junction using forward vector primers F1-6 paired with the reverse primer located in *cak1* gene. Primer sequences are given in Table S1.

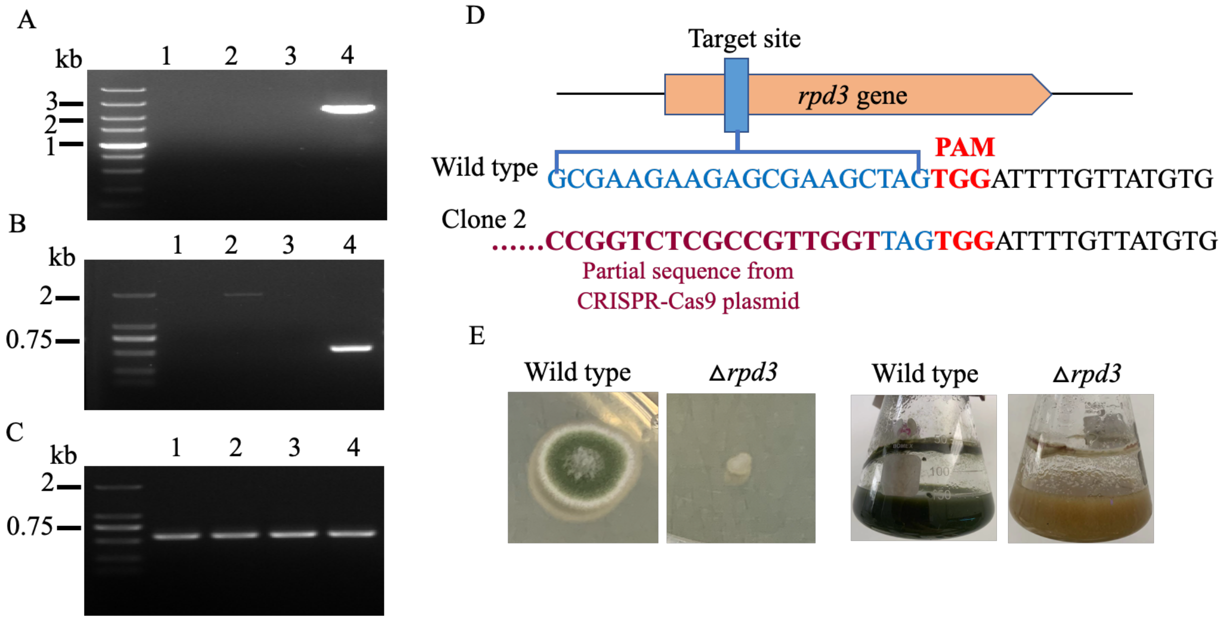

Figure S8. Inactivation of the *rpd3* gene in *Aspergillus* sp. SCSIO SX7S7 using CRISPR-Cas9. (A) DNA amplification of the DNA regions surrounding the full *rpd3* gene of the three clones picked out from the regeneration plate using primers flanking the *rpd3* gene. (B) DNA amplification of the DNA regions surrounding the PAM site of the three clones picked out from the regeneration plate using primers flanking the PAM site. (C) Positive control for checking the DNA template quality of the clones picked out from the regeneration plate using primers flanking the ITS region. (D) Sequence analysis of PCR products of clone 2. (E) Morphological comparison between the representative Δ*rpd3* mutant and 7S7 wild type strains on complete plate at 28 °C for 4 days (left panel) and on liquid PDB medium (right panel). (A–C, lane 1-3: tested *rpd3* mutation clones; lane 4: wild type strain)

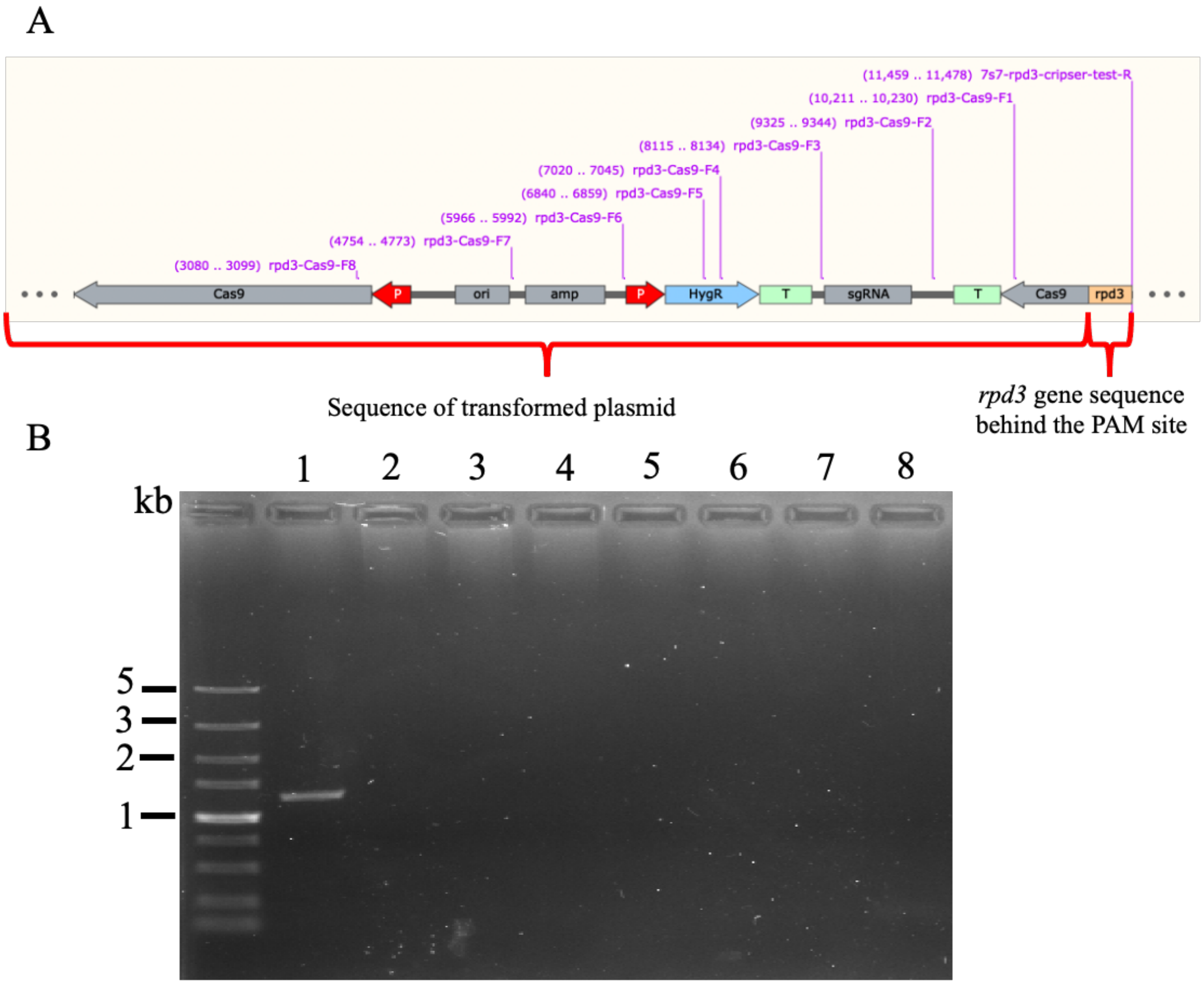

Figure S9. PCR verification of the inserted pBSKII-to*Cas9*-*hph*-sgRNA plasmid sequence into *rpd3* gene. To test the validity of the insertion sequence, DNA amplifications were performed across the insert-genomic DNA junction using flanking genomic DNA primer “R” and vector primers “F1-8” (A) . Only one PCR products with expected size was observed using vector primer F1 and *rpd3* gene primer R, while all other vector primers (F2-8) failed to get any PCR band (B), suggesting the intervening vector sequence might be rearranged. Lane 1-8: DNA amplifications of vector-*rpd3* junction using forward vector primers F1-8 paired with the reverse primer located in *rpd3* gene. Primer sequences are given in Table S1.

Table S1. Strains and plasmids used in this study

Strians/plasmids	Characteristic(s)	Source
Spiromastix sp. SCSIO F190	Wild-type strain, spiromarmycin producing	Ref [26]
Aspergillus sp. SCSIO SX7S7	Wild-type strain, bioactive secondary metabolites producing	Ref [27]
Δ*creA*-F190	Δ*creA*::*hph* in F190 WT	This study
Δ*cak1-7S7*	Δ*cak1*::*hph* in SX7S7 WT	This study
Δ*rpd3-7S7*	Δ*rpd3*::*hph* in SX7S7 WT	This study
pBSKII-*toCas9-hph*	pBSKII-PtrPC-Flag-*toCas9*-TtrPC containing the *hph* cassette	Ref [25]
pFC330	sgRNA expression cassette	Ref [16]
pBSKII-to*Cas9*-*hph*-sgRNA	Cas9 and sgRNA expression vector	This study

Table S2. Primers used in this study

Name	Sequence (5' to 3')	Experiment
Frag1-F	tcagggcgatggcccactacgcgtaagctccctaattggc	Cloning of sgRNA expression cassette
Frag2-R	tgattagggtgatggttcacgagccaagagcggattcct cagtctcgta	Cloning of sgRNA expression cassette
f190- *creA* -cripser-frag1-R	gacgagcttactcgtttcgtcctcacggactcatcaggt cgcacggtgat	Cloning of *creA* sgRNA expression cassette
f190- *creA* -cripser-frag2-F	acgagtaagctcgtcgtcgcagagaggacacttgtg ttttagagctagaa	Cloning of *creA* sgRNA expression cassette
7s7-*cak1*-cripser-frag1-R	gacgagcttactcgtttcgtcctcacggactcatcaggacc ggcggtgat	Cloning of *cak1* sgRNA expression cassette
7s7-*cak1*-cripser-frag2-F	acgagtaagctcgtcgaccggagatgcctcgaatggtttt agagctagaa	Cloning of *cak1* sgRNA expression cassette
7s7-*rpd3*-cripser-frag1-R	gacgagcttactcgtttcgtcctcacggactcatcagg cgaagcggtgatgtctg	Cloning of *rpd3* sgRNA expression cassette
7s7-*rpd3*-cripser-frag1-F	acgagtaagctcgtcgcgaagaagagcgaagc taggttttagagctagaaatagc	Cloning of *rpd3* sgRNA expression cassette
creA-deletion-test-F	tggatttctccaacctcctg	Amplification of the DNA region surrounding the PAM site of *creA*
creA -deletion-test-R	tgtcgagtttggtgacgaag	Amplification of the DNA region surrounding the PAM site of *creA*
F190- *creA* -full-test-F	cgtcactcaacagcaccaat	Amplification of the full *creA* gene
F190- *creA* -full-test-R	ccaagccaaacccaaacatg	Amplification of the full *creA* gene
7s7-*cak1*-cripser-test-F	gaccggagatgcctcgaatg	Amplification of the DNA region surrounding the PAM site of *cak1*
7s7-*cak1*-cripser-test-R	tcagcagcctcagcctctat	Amplification of the DNA region surrounding the PAM site of *cak1*
7s7-*cak1*-full-test-F	ttactctgattgcctccagc	Amplification of the full *cak1* gene
7s7-*cak1*-full-test-R	tagtcgggacttaccaacga	Amplification of the full *cak1* gene
7s7-*rpd3*-cripser-test-F	gcgaagaagagcgaagctag	Amplification of the DNA region surrounding the PAM site of *rpd3*
7s7-*rpd3*-cripser-test-R	ttaacgcagtttgcgtgacc	Amplification of the DNA region surrounding the PAM site of *rpd3*
7s7-*rpd3*-full-test-F	tcaccttttgctcggctctc	Amplification of the full *rpd3* gene
7s7-*rpd3*-full-test-R	gaccctcctaaagctatgcc	Amplification of the full *rpd3* gene
ITS1	tccgtaggtgaacctgcgg	Amplification of the ITS region
ITS4	tcctccgcttattgatatgc	Amplification of the ITS region

cak1-Cas9-F1	ccggagttgagacaaatggt	Amplification of insert-genomic DNA junction
cak1-Cas9-F2	aaggccaccgccaagtactt	Amplification of insert-genomic DNA junction in *cak1* gene
cak1-Cas9-F3	aagaacggctacgccggcta	Amplification of insert-genomic DNA junction in *cak1* gene
cak1-Cas9-F4	gcgagtcagtgagcgaggaa	Amplification of insert-genomic DNA junction in *cak1* gene
cak1-Cas9-F5	cgcggaacccctatttgttt	Amplification of insert-genomic DNA junction in *cak1* gene
cak1-Cas9-F6	cctttgacgttggagtccac	Amplification of insert-genomic DNA junction in *cak1* gene
rpd3-Cas9-F1	cgatcgtggtgtcaaagtac	Amplification of insert-genomic DNA junction in *rpd3* gene
rpd3-Cas9-F2	attcgccattcaggctgcgc	Amplification of insert-genomic DNA junction in *rpd3* gene
rpd3-Cas9-F3	gaaaaaccgtctatcagggc	Amplification of insert-genomic DNA junction in *rpd3* gene
rpd3-Cas9-F4	atttcggctccaacaatgtcctgacg	Amplification of insert-genomic DNA junction in *rpd3* gene
rpd3-Cas9-F5	cgcaaggaatcggtcaatac	Amplification of insert-genomic DNA junction in *rpd3* gene
rpd3-Cas9-F6	ccaataggccgaaatcggcaaaatccc	Amplification of insert-genomic DNA junction in *rpd3* gene
rpd3-Cas9-F7	tttctacggggtctgacgct	Amplification of insert-genomic DNA junction in *rpd3* gene
rpd3-Cas9-F8	gtgttgcccaggaccttaaa	Amplification of insert-genomic DNA junction in *rpd3* gene
